# New freshwater *Ceratomyxa* species, *Ceratomyxa affinis* n. sp. (Myxozoa: Ceratomyxidae) in the gallbladder of *Leporinus affinis* from Brazilian Amazon

**DOI:** 10.1590/S1984-29612025054

**Published:** 2025-10-17

**Authors:** Luize Cristine Pantoja dos Reis, Abthyllane Amaral de Carvalho, Roger Leomar da Silva Ferreira, Saturo Cardoso Morais, Igor William Couto Souza, Rafaela Franco de Araujo, Marcela Nunes Videira

**Affiliations:** 1 Universidade Federal do Amapá – UNIFAP, Programa de Pós-graduação em Ciências Ambientais, Macapá, AP, Brasil; 2 Universidade Federal do Pará – UFPA, Instituto de Ciências Biológicas, Programa de Pós-graduação em Biologia de Agentes Infecciosos e Parasitários, Belém, PA, Brasil

**Keywords:** Myxozoan, fish, parasite, Amazon, parasitology, Myxozoários, peixe, parasito, Amazônia, parasitologia

## Abstract

The genus *Ceratomyxa* is composed of approximately 300 described species, most of which are found parasitizing hosts in marine aquatic environments. The present study, through phylogenetic, molecular and morphological analyses, described a new species of *Ceratomyxa* found parasitizing *Leporinus affinis* specimens from the Tartarugalzinho River, in municipality of Tartarugalzinho, state of Amapá, Brazil. The new species was found parasitizing the urinary bladder of *L. affinis*. The myxospores of *Ceratomyxa affinis* n. sp. are 7.2 µm long and 43.2 µm thick with a posterior angle of 170º. The polar capsules measure 3.9 µm long and 4.05 µm wide. Based on morpho-molecular analyses, it was possible to prove that *Ceratomyxa affinis* n. sp. is a new species, contributing to the knowledge of the parasitic fauna of fish in the Amazon region.

## Resumo

O gênero *Ceratomyxa* é composto por cerca de 300 espécies descritas encontradas em sua maioria parasitando hospedeiros de ambientes aquáticos marinhos. O presente estudo, usando análises filogenéticas, moleculares e morfológicas, descreveu um uma nova espécie de *Ceratomyxa* encontrado parasitando *Leporinus affinis* oriundos do Rio Tartarugalzinho, município de Tartarugalzinho, no estado do Amapá, Brasil. A nova espécie foi encontrada parasitando a vesícula urinária de *L. affinis.* Os myxosporos de *Ceratomyxa affinis* n. sp. possuem 7.2 µm de comprimento e 43.2 µm de espessura com ângulo posterior de 170º. As capsulas polares medem 3.9 µm de comprimento e 4.05 µm de largura. Baseada nas análises morfo-molecular, foi possível comprovar que *Ceratomyxa affinis* n. sp. é uma nova espécie, contribuindo para o conhecimento da parasitofauna de peixes da região amazônica.

## Introduction

Parasites belonging to the class Myxozoa are classified as obligate eukaryotic endoparasites and present a complex life cycle that involves vertebrate and invertebrate hosts. This group is considered one of the most abundant in terms of species richness, with approximately 2,600 described species, frequently found in aquatic hosts. Myxozoan spores have simple morphological structures, with a rounded, oval or ellipsoid-shaped body, which has two valves that surround two polar capsules, which in some cases have different sizes. (Atkinson et al., 2018; Okamura et al., 2018; Sindeaux-Neto, et al., 2021; Eiras et al., 2023; Cardoso et al., 2025).

The genus *Ceratomyxa* Thélohan, 1892 is composed of about 300 described species that are mostly found parasitizing hosts in marine aquatic environments. However, there are also records of species described parasitizing freshwater fish mainly, in South America. Myxospores of this genus exhibit an elongated or arcuate-shaped spore body with two polar capsules located at the top of the spore, parallel to the suture line, and they are commonly found parasitizing the host's gallbladder (Zatti et al., 2018, 2023 ; Eiras et al., 2018; Martel et al., 2024; Carvalho et al., 2024).

Among the fish that make up the Anastomidae family, there is the genus *Leporinus* Agassiz 1829, which houses the largest number of described species, and is widely distributed in all river basins in the country. The fish belonging to this group are of great commercial importance and are popularly known in Brazil as “aracus” and “piaus.” *Leporinus affinis* Günther 1864 is an anastomid belonging to the genus *Leporinus*, known as “piau-flamengo”. Specimens of this species have a fusiform body structure with a coloration composed of dark and transversal bars on the body and can reach 30 cm in total length. These fish have herbivorous habits and are commonly found in streams with rocky and sandy bottoms. (Santos & Zuanon, 2008; Birindelli & Britski, 2013; Zacardi et al., 2017; Froese & Pauly, 2024).

Studies focused on the parasitic fauna of fish are of utmost importance as they can be used to understand the functioning of the parasite-host-environment ecological triad. In the context of parasites infecting aquatic animals, fish found in lakes and rivers are the most common vectors for a wide variety of parasitic organisms, with myxozoa being one of the most common. With that in mind, therefore, the present study uses phylogenetic, molecular and morphological analyses, to describe a new species of *Ceratomyxa* found parasitizing the gallbladder of specimens of *L. affinis* from the Tartarugalzinho River, Municipality of Tartarugalzinho, state of Amapá, in eastern Amazon.

## Material and Methods

### Study area and fish collection

**Figure 1:**
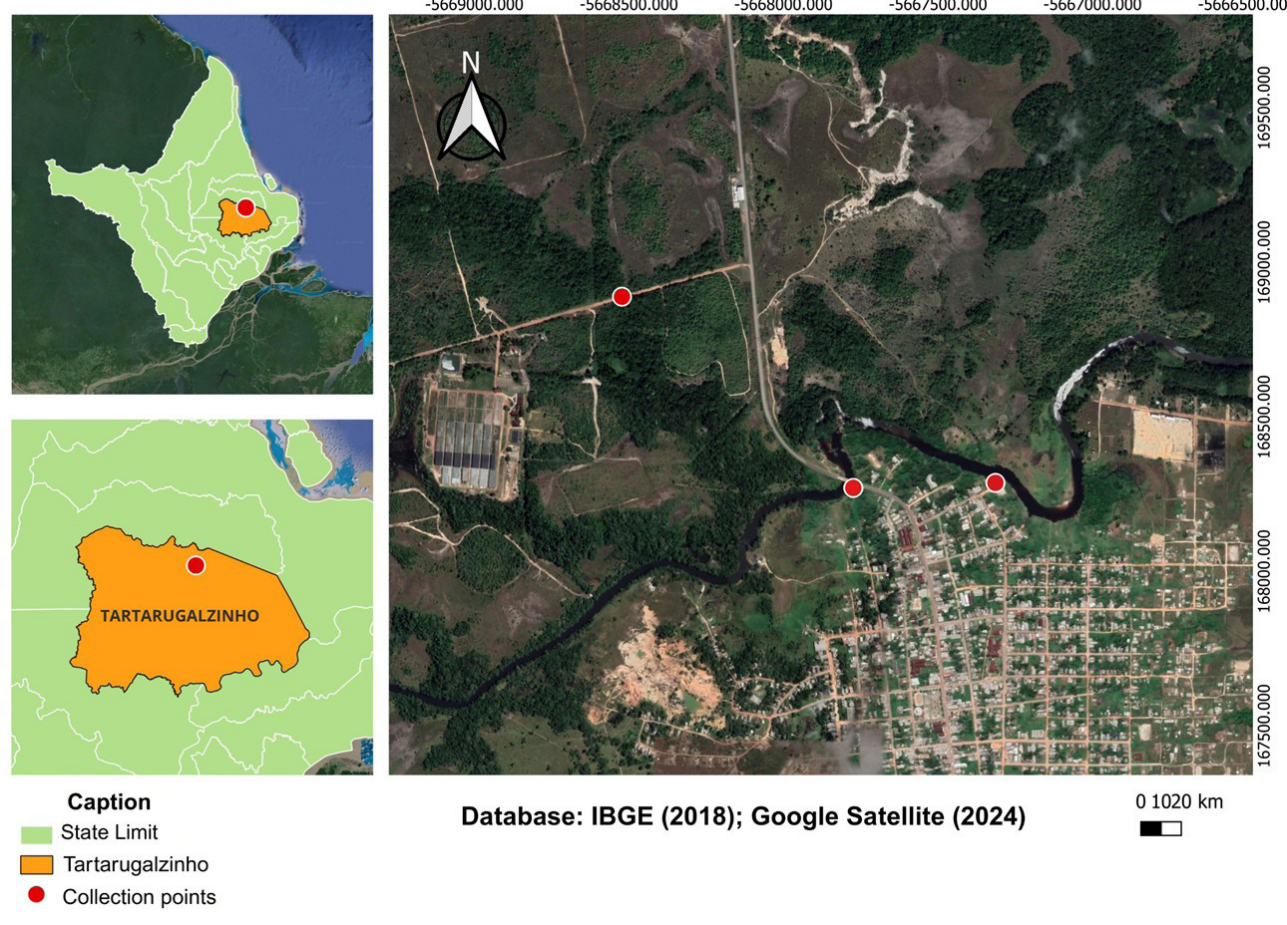
Location map of *Leporinus affinis* collections in Tartarugalzinho River (red dots), in municipality of Tartarugalzinho, state of Amapá: Pedreira River, Brazilian Amazon.

The municipality of Tartarugalzinho is an important component of the Amapá fishing sector. It is part of a lake region and has an extensive biodiversity found in its vegetation, waterfalls, lakes and rivers. The Tartarugalzinho River (Figure 1) is the most important river in the municipality. Draining art of its waters into the Duas Bocas Lake, it has extensive vegetation cover and a unique ecosystem that enables a rich ichthyofauna (Bidone et al., 1997; Yokomizo, 2012; Silveira et al., 2015).

The specimens of *Leporinus affinis* (n= 13) were collected between August 14, 2023 and October 9, 2024. The specimens were collected by the team from the Laboratory of Morphophysiology and Animal Health at the State University of Amapá (LABMORSA) with the help of a local fisher. After being collected, the specimens were transported alive to the Laboratory of Morphophysiology and Animal Health (LABMORSA), in thermal boxes containing water from the environment itself and artificial aeration. The collections were approved by the Animal Use Committee (CEUA) and registered in the Biodiversity Authorization and Information System (SISBIO/ICMBIO).

### Dissection of host fish

In LABMORSA, the fish were anesthetized with tricaine methanesulfonate (MS222 Sigma), and subsequently desensitized by means of neural myelotomy with the aid of sharp forceps. To measure the biometric data, the specimens were weighed (g) and measured (cm). With the anesthetized specimens, macroscopic analysis was performed on the animal's body surface, under a binocular stereomicroscope, with the aim of observing the existence of cysts in the tegument, lesions or loss of lining. Then, an incision was made in the ventral region of the specimens so that the internal organs were exposed. Afterwards, with the help of sharp forceps, the gallbladder was removed without leaking the internal liquid, placed between a slide and a coverslip and observed fresh using light microscopy.

### Morphometry and histological analysis

All gallbladder that showed the presence of parasites were placed between slide and coverslip and photographed using a digital camera (Moticam 2300 3.0 M), for fresh morphological observation of the parasite. The analysis of the morphometric characteristics of the spores found was performed according to the methodology proposed by Lom & Arthur (1989), and consisted of spore length (SL), spore thickness (ST), polar capsule length (PCL), polar capsule width (PCW) and posterior angle (PA), dimensions were obtained by averaging all measurements of 30 spores. The calculation of parasite prevalence was tabulated according to Bush et al. (1997).

The fragments of gallbladder parasitized were collected and fixed in Davidson (95% alcohol, formaldehyde, acetic acid and water) for a period of 24 h and subsequently dehydrated in an increasing battery of alcohols (70%, 80%, 90%, 100% I, 100% II and 100% III), and were then subjected to diaphanization in xylene and impregnated in paraffin blocks. After cooling, the blocks were cut by a microtome into 5 µm-thick ribbons. The cut material was fixed on a glass slide and stained with hematoxylin-eosin (H&E) (Luna, 1968).

### DNA extraction and sequencing

To perform molecular biology, gallbladder infected with microparasite were collected and stored in Eppendorf tubes with 80% ethyl alcohol. DNA from each sample was extracted using the ReliaPrep gDNA Tissue Miniprep System kit (Promega, USA) according to the protocol provided by the manufacturer. The samples were sized by spectrometry (Biodro Duo) with a wavelength of 250nm; for the amplification of the 18sDNA gene fragment the polymerase chain reaction (PCR) was performed in a thermocycler (MyGene MG96G).

The primers 18E (5'-CTGGTTGATCCTGCCAGT-3') (Hillis & Dixon, 1991) and 18R (5'- CTACGGAAACCTGTTACG-3') (Whipps et al., 2003) were used for the first stage of amplification. Cycling was performed under the following conditions: initial denaturation at 95°C for 15 min, followed by 35 cycles of 95°C for 1 min, 48°C for 1.5 min, 72°C for 2 min and final extension at 72°C for 10 min. For the second round of amplification, primers 18E - MC3 (5'-GATTAGCCTGACGATCACTCCACGA-3') and 18R-MC5 were used (5'-CCTGAGAAACGGCTACCACATCCA-3') (Molnár et al., 2002). The cycle with initial denaturation at 95°C for 15 min was applied, followed by 35 cycles of 95°C for 30 seconds, 56°C for 30 seconds, 72°C for 1 min andmin and the final extension at 72°C for 10 min.

The PCR results were subjected to electrophoin resis 1.5% agarose gel in a Tris-borate-EDTA (TBE) buffer solution, then stained with UniSafe Dye (UniScience, Brazil) and subsequently visualized using the Bluegel Electrophoresis System. All samples that presented positive results were sent to ACTGene (Alvorada, RS, Brazil) for the sequencing process.

### Phylogenetic analyses

The sequences obtained were edited in the Geneious^®^ 7.1.3 software. After BLASTn searches (Altschul et al., 1997), the result generated was compared with sequences deposited in GenBank through the Basic Local Alignment Search Tool (BLASTn) of the National Center for Biotechnology Information (NCBI), to determine the similarity between the sequences.

A database was aligned using the ClustalW algorithm with its default parameters, in the Geneious 7.1.3 software (Kearse et al., 2012). Maximum parsimony Bayesian inference (BI) analyses were performed, with the aid of the MrBayes software 3.2.7a (Ronquist & Huelsenbeck, 2003), through the CIPRES platform based on the evolution model (GTR + I + G), chosen by the jModelTest evaluation, based on the lowest classification of the Bayesian information criterion (BIC). The following probabilities were based on 10 million generations using Markov Chain Monte Carlo algorithms (MCMC). A tree was generated from the BI products and was evaluated using the topologies (Miller et al., 2010). With the help of the MEGA11 program, it was possible to observe the genetic distance of myxozoans and thus define the connections between species. FigTree 1.3.1 software was used to generate the tree and CorelDraw 2019 was used to adjust and format it.

## Results

### Morphological description of spores

**Figure 2:**
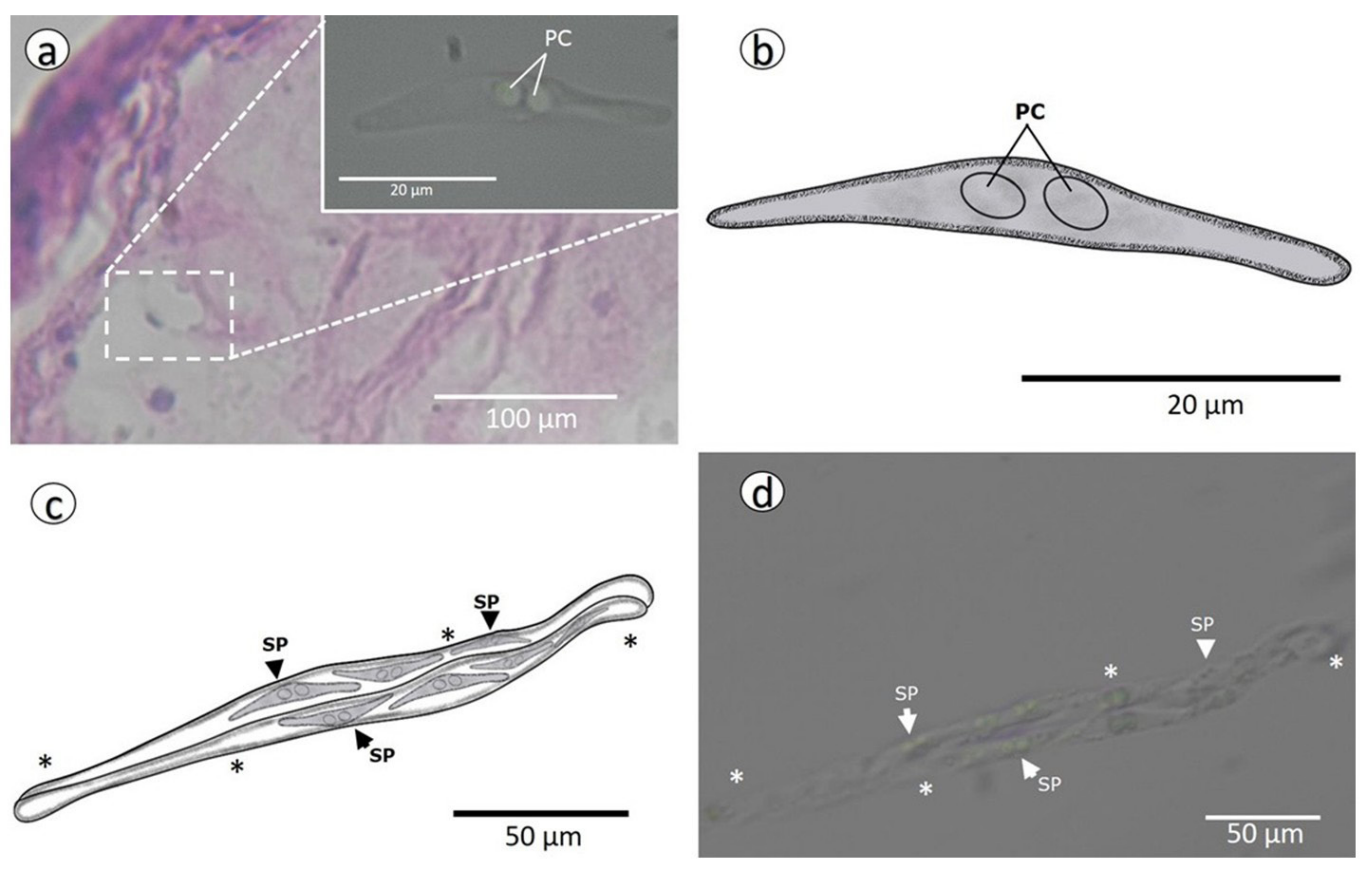
**(**A) Histological section of the fish gallbladder showing the spores of *Ceratomyxa affinis* n. sp. stained with eosin and hematoxylin. Highlighting: Single myxopore of *Ceratomyxa affinis* n. sp.; (B) Schematic drawing of *Ceratomyxa affinis* n. sp.; (C) Schematic drawing of plasmodium of *Ceratomyxa affinis* n. sp.; (D) Photomicrography of plasmodium (*) with fresh spores *of Ceratomyxa affinis* n. sp., in the gallbladder of *Leporinus affinis*. PC: polar capsule; SP: spore.

Thirteen specimens of *L. affinis* were examined, of which 10 (76.9%) were parasitized by *Ceratomyxa affinis* n. sp., present in plasmodia in the gallbladder and as loose myxospores in the bile. The plasmodia were elongated and vermiform-like (Figures 2C-D), and the myxospores were slightly arched (Figures 2A-B) and had rounded ends.

The myxospores of *Ceratomyxa affinis* n. sp. are 7.2 µm long and 43.2 µm thick with a posterior angle of 170º. The polar capsules are 3.9 µm long and 4.05 µm wide. Due to the conditions of the processed material, it was not possible to observe the turns of the polar filament. Regarding the motility of the plasmodia, they had undulating movements when observed fresh under light microscopy.

### Remarks

In the state of Amapá, five species of *Ceratomyxa* spp. have been described, namely *Ceratomyxa gracillima* Zatti et al., 2018, *Ceratomyxa macapaensis* Bittencourt et al., 2022, *Ceratomyxa matosi* Martel et al., 2024, *Ceratomyxa edilsonis* Carvalho et al., 2024, *Ceratomyxa tavariensis* Araújo et al., 2024 and *Ceratomyxa tessaloniensis* Cardoso et al., 2025 in *Mesonauta festivus*, *Boulengerella cuvieri*, *Pimelodella cristata*, *Pterophyllum scalare* and *Astyanax mexicanus*, respectively. This makes *Ceratomyxa affinis* n. sp. described in *L. affinis* the seventh species of the genus *Ceratomyxa* occurring in the state of Amapá and the third species described from the Tartarugalzinho River, in the municipality of Tartarugalzinho.

When comparing the morphometric data of the myxosporean *Ceratomyxa affinis* n. sp. with the other species of *Ceratomyxa* spp. described in the state of Amapá, it is possible to observe that all the measurements of *Ceratomyxa affinis* n. sp. are larger than for all *Ceratomyxa* spp. described not only in the state of Amapá, but also in the Amazon region. Regarding the motility of the plasmodia and myxospore formation of *Ceratomyxa* spp. described in Amapá, the plasmodia and myxospores of *Ceratomyxa affinis* n. sp. resemble those described for *C. edilsonis, C. matosi, C. tavariensis* and *C. macapaensis*, which present vermiform plasmodia and slightly arched myxospores. This differs from the plasmodia of *C. tessaloniensis*, which had amoeboid-like motility and strongly arched myxospores.

### Taxonomic summary

Kingdom: Animalia Linnaeus, 1758

Class Myxozoa Grassé, 1970 (Kyger et al., 2021)

Subclass Myxosporea Bütschli, 1881

Order Bivalvulida Shulman, 195

Family Ceratomyxidae Doflein, 1899

Genus *Ceratomyxa* Thélohan, 1892

Species Ceratomyxa affinis n.sp.

**Host:***Leporinus affinis* Günther 1864

**Site of infection:** Gallbladder

**Prevalence:** 10/13 (76.9%)

**Locality:** Tartarugalzinho River, municipality of Tartarugalzinho, state of Amapá, Brazil (01º30’32.5” N; 050º55’10.3” W)

**Type material:** Glass slide with spores stained with Hematoxylin and Eosin (H&E) was deposited in the collection of the National Amazonas Research Institute (INPA), Manaus, Amazonas, Brazil (accession number: CND 000111).

**GenBank accession number:** PV765332

**Etymology:** The species-specific epithet refers to the species name of the host fish.

### Molecular data and phylogenetic analysis

The partial SSU rDNA sequence for *Ceratomyxa affinis* n. sp. obtained in the present study had 870 base pairs (GenBank accession number: PV765332), which were G + C (A = 0.2718, C = 0.2007, G = 0.2849, T = 0.2426). Assuming a GTR + G model of nucleotide substitution, estimated nucleotide substitution rates were A - C = 0, 9048, A - G = 2.1973, A - T = 1.4964, C - G = 0.6508, C - T = 3.8475, G - T = 1.0000, with a gamma distribution of G = 0.5390.

To construct the phylogenetic tree, 16 sequences of species from the Ceratomyxidae family that are available in GenBank were used, and *Ellipsomyxa tucujuensis* Ferreira et al., 2021 and *Myxodavisia bulani* (Fiala et al., 2015) were used as outgroups. BLASTn search showed that the sequence of *Ceratomytxa affinis* n. sp. did not match any other sequence deposited in GenBank. In this analysis, no sequence had greater than 85.25% similarity with *Ceratomyxa affinis* n. sp. The analysis of the degree of coverage in BLASTn with the SSU 18S rDNA analysis from other *Ceratomyxa* spp., showed an approximate average similarity of 99% with the target sequence.

The analysis of the *p* distance showed a large genetic divergence between the other species of *Ceratomyxa* spp. (Table 1). The smallest genetic distance (*p*) found between *Ceratomyxa affinis* n. sp. with another species of *Ceratomyxa* was 6.8% with *Ceratomyxa vermiformis* Adriano and Okamura, 2017 and no more than 10.7% with *Ceratomyxa mandii* Araujo et al., 2022 (Table 1).

**Table 1:** Comparative genetic distance (p) recorded among Ceratomyxidae

**Species**	**(1)**	**(2)**	**(3)**	**(4)**	**(5)**	**(6)**	**(7)**	**(8)**	**(9)**
(1) *Ceratomyxa affinis* n. sp.	*-*	-	-	-	-	-	-	-	-
(2) *C. tavariensis*	0.101	-	-	-	-	-	-	-	-
(3) *C. amazonensis*	0.099	0.012	-	-	-	-	-	-	-
(4) *C. brasiliensis*	0.099	0.034	0.036	-	-	-	-	-	-
(5) *C. ranunculiformis*	0.100	0.027	0.032	0.024	-	-	-	-	-
(6) *C. barbata*	0.086	0.077	0.077	0.079	0.075	-	-	-	-
(7) *C. macapaensis*	0.081	0.058	0.060	0.065	0.058	0.052	-	-	-
(8) *C. vermiformis*	**0.068** *	0.063	0.060	0.065	0.060	0.045	0.010	-	-
(9) *C. fonsecai*	0.094	0.091	0.085	0.092	0.094	0.079	0.078	0.071	-
(10) *C. mandii*	0.107	0.102	0.101	0.102	0.099	0.101	0.079	0.074	0.092

* Smallest distance.

**Figure 3:**
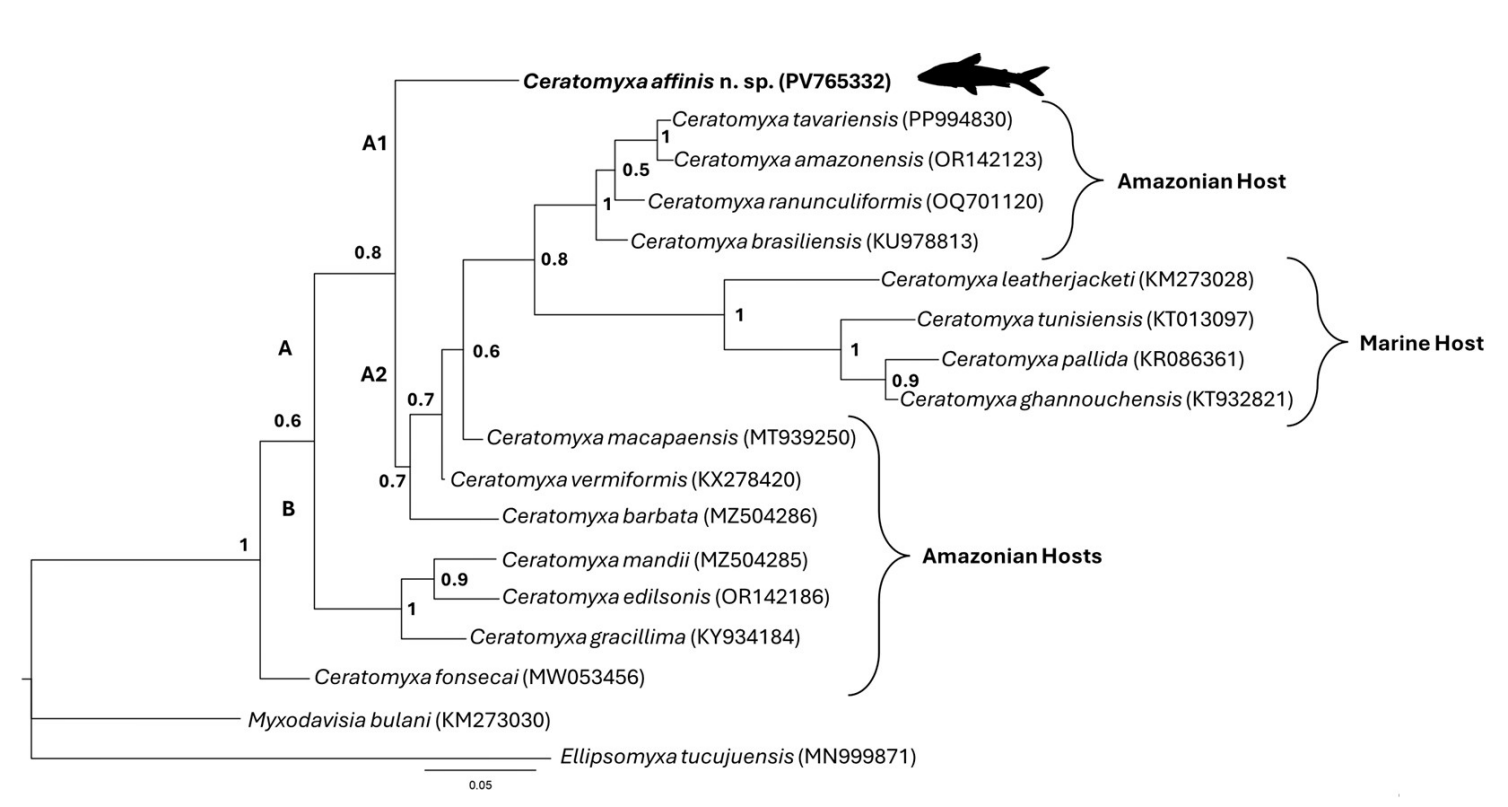
Maximum likelihood phylogenetic tree based on SSU-rDNA sequences of *Ceratomyxa affinis* n. sp. and other myxozoans. Nodal supports are indicated for Bayesian inference with posterior probabilities. GenBank accession numbers are presented after each myxozoans species name.

The phylogenetic tree showed two main clades, A and B, as shown in Figure 3, with strong nodal support. Clade A was formed by hosts found in the Brazilian Amazon and some marine species, whereas Clade B was formed by species that parasitized hosts of the order Siluriforms from the Brazilian Amazon region.

*Ceratomyxa affinis n*. sp. did not group with any species of *Ceratomyxa* spp. in clade A1 (nodal support = 0.8), while in clade A2 (nodal support = 0.7) all other species of *Ceratomyxa* spp. were organized, from both the Amazon region and from the marine environment. In the phylogenetic arrangement presented in Figure 3, a species of *Ceratomyxa*, *Ceratomyxa ALTERADO* Silva et al., 2020, described in *Hemiodus unimaculatus* Bloch, 1794, grouped in isolation, showing the same behavior as *Ceratomyxa affinis* n. sp. in clade A1.

## Discussion

The genus *Ceratomyxa* is characterized by slightly arched myxospores, with two elongated valves that are generally wider than they are long (Gunter et al., 2009). Normally, myxospores of this genus are found inside plasmodia, and may have a shape and movement similar to worms or amoeboids (Zatti et al., 2023; Müller et al., 2025).

The myxospores of *Ceratomyxa affinis* n. sp. were found in vermiform plasmodia, as were the myxospores of *C. edilsonis* and C. *tavariensis* described in *P. cristata* and *P. scalare*, respectively (Araújo et al., 2024; Carvalho et al., 2024). Furthermore, the aforementioned species of *Ceratomyxa* spp. were captured in the same study area of this work, in the Tartarugalzinho River. There is thus a pattern of Ceratomyxidae plasmodia for the studied area. The plasmodia of *Ceratomyxa affinis* n. sp. differ from the plasmodia of *C. tessaloniensis* found in *A. mexicanus*, in the Flexal River, in the municipality of Macapá, which had movement and had amoeboid-like shapes. (Cardoso et al., 2025).

Müller et al. (2025) state that the motility of *Ceratomyxa* spp. plasmodia is restricted to the freshwater lineage of South America and to a few marine species, and that there is a large gap in the observation of the vegetative stages of coelozoic Ceratomyxidae, since the plasmodia structures are delicate and easily degraded post-mortem in the host, leaving only free myxospores in the parasitized organ.

Adriano et al. (2021) state that plasmodia of *Ceratomyxa* spp. with undulating motility similar to nematodes are exclusive to freshwater, that is, the plasmodia of *Ceratomyxa affinis* n. sp. confirm what was previously described, as also happens with the plasmodia of *C. macapensis* in *M. festivus* (Bittencourt et al., 2022), *Ceratomyxa fonsecai* in *Hemiodus unimaculatus* (Silva et al., 2020), *C. matosi* in *B. cuvieri* (Martel et al., 2024) and *Ceratomyxa raniculiformis* in *Plagioscion squamosissimus* (Zatti et al., 2023). All species of *Ceratomyxa* spp. from the Amazon region have polysporic plasmodia, as does *Ceratomyxa affinis* n. sp., which according to Silva et al. (2020), Okamura et al. (2015) and Bartošová-Sojková et al. (2018), is considered a more efficient strategy for dispersing parasites in the environment.

In the Amazon region, all species of *Ceratomyxa* spp. were found only in the gallbladder of their hosts, meaning that they showed tropism for this organ, a characteristic for freshwater fish. Nonetheless, there a report of the occurrence of *Ceratomyxa* spp. in the urinary bladder of marine fish (Eiras, 2006; Eiras et al., 2018).

Regarding the morphometric measurements of the myxospores of *Ceratomyxa affinis* n. sp., it was possible to observe that this presents the largest measurements of myxospores of *Ceratomyxa* spp. in the Amazon region. The myxospore length of *Ceratomyxa affinis* n. sp. (7.2 µm) was close to the length of *Ceratomyxa amazonensis* Mathews et al., 2016 with 7.0 µm. All other measurements were not close to any other species of *Ceratomyxa* spp. described in the Brazilian Amazon region (Table 2).

**Table 2:** Comparative morphometric table of measurements (µm) of *Ceratomyxa affinis* n. sp. with other species of *Ceratomyxa* spp. described in brazilian amazon.

**Species**	**Myxopore dimensions (µm)**							**Myxospore shape**	**Plasmodia motility**	**Host**	**Locality**	**Reference**	**BP**
	**ML**	**MT**	**Ratio**	**PCL**	**PCW**	**PA**	**Coils**						
*Ceratomyxa affinis* n. sp.	7.2	43.2	6.0	3.9	4.05	170º	-	Lightly arcuate	Vermiform- like	*Leporinus affinis*	Tartarugalzinho River-AP	This study	870
*C. tessaloniensis*	3.13	12.18	3.89	1.74±0.1	1.5±0.16	74º	-	Strongly arcuate	Amoeboid-like	*Astyanax mexicanus*	Flexal River-AP	Cardoso et al. (2025)	932
*C. deformis*	3.9	35.7	-	1.8	1.4	167º	4	Lightly arcuate	Amoeboid-like	*Schyzodon fasciatus*	Amazon and Tapajós River-PA	Müller et al. (2025)	1599
*C. anomala*	3.5	20.4	-	1.8	1.3	133º	4	Lightly arcuate	Amoeboid-like	*Schyzodon fasciatus*	Amazon and Tapajós River-PA	Müller et al. (2025)	1466
*C. tavariensis*	1.6	11.5	-	0.7	0.6	124º	3-4	Lightly arcuate	Vermiform- like	*Pterophyllum scalare*	Tartarugalzinho River-AP	Araújo et al. (2024)	812
*C. edilsonis*	1.64	17.13	-	1.36	0.9	152º	4-5	Lightly arcuate	Vermiform- like	*Pimelodella cristata*	Tartarugalzinho River-AP	Carvalho et al. (2024)	922
*C. matosi*	5.2	24.5	-	2.2	2.2	-	4-5	Lightly arcuate	Vermiform- like	*Boulengerella cuvieri*	Reservoir of HPP Coaracy Nunes-AP	Martel et al. (2024)	560
*C. raniculiformis*	4.9	37.6	-	2.0	1.9	165º	2-3	Elongated/lightly arcuate	Amoeboid-like	*Plagioscion squamosissimus*	Lago Grande do Curuai-PA	Zatti et al., 2023	2044
*C. barbata*	2.9	21.7	-	1.6	1.4	164º	3	Elongated/lightly arcuate	Vermiform-like	*Rhaphiodon vulpinus*	Tapajós River-PA	Franzolin et al. (2022)	1448
*C. mandii*	4.6	31.2	-	1.8	1.9	162º	3-4	Elongated/lightly arcuate	Vermiform-like	*Pimelodina flavipinnis*	Solimões River-AM	Araújo et al. (2022)	1546
*C. macapaensis*	4.2	22.7	5.41	1.86	1.6	-	3-4	Elongated/lightly arcuate	Vermiform-like	*Mesonauta festivus*	Piririm River-AP	Bittencourt et al. (2022)	810
*C. fonsecai*	2.6	28.9	11.12	1.9	1.7	164.8º	3-4	Elongated/lightly arcuate	Vermiform-like	*Hemiodus unimaculatus*	Tocantins River-MA	Silva et al. (2020)	1449
*C. gracillima*	4.4	7.0	6.36	1.9	-	37º	2-3	Strongly arcuate	Vermiform-like	*Brachyplatystoma rousseauxii*	Tapajós, Amazon and Solimões River-PA	Zatti el al. (2018)	1849
*C. brasiliensis*	6.3	41.2	6.54	2.6	2.5	147º	3-4	Elongated/lightly arcuate	Vermiform-like	*Cichla monoculus*	Tapajós River-PA	Zatti et al. (2017)	1605
*C. vermiformis*	4.5	8.4	5.24	2.7	-	30.2	3-4	Strongly arcuate	Vermiform-like	*Colossoma macropomum*	Amazon and Solimões River-PA	Adriano & Okamura, (2017)	1601
*C. amazonensis*	7.0	15.8	2.24	3.2	3.6	103.7º	3-4	Arcuate	-	*Symphysodon discus*	Negro River-AM	Mathews et al. (2016)	1527
*C. microlepis*	5.2	35.5	6.83	2.2	-	162.3	5-6	Elongated/lightly arcuate	-	*Hemiodus microlepis*	Trombetas River-PA	Azevedo et al. (2013)	-

ML: myxospore length; MT: myxospore thickness; PCL: Polar capsule length; PCW: Polar capsule width; PA: posterior angle; BP: Number of base pairs of *Ceratomyxa* species.

The shape of the myxospores of *Ceratomyxa affinis* n. sp. was slightly arched, as was also the case with *Ceratomyxa deformis*, *Ceratomyxa anomala*, *C. matosi, C. macapaensis, C. edilsonis* and *C. tavariensis* (Müller et al., 2025; Martel et al., 2024; Carvalho et al., 2024; Araújo et al., 2024). In South America, only three species of *Ceratomyxa* spp. have strongly aqueous myxospores, these being *Ceratomyxa vermiformis* in *Colossoma macropomum* (Adriano & Okamura, 2017), *Ceratomyxa gracillima* in *Brachyplatystoma rousseauxii* (Zatti et al., 2018) and *C. tessaloniensis* in *A. mexicanus* (Cardoso et al., 2025).

According to Fiala et al. (2015), *Ceratomyxa leatherjacketi* and *Ceratomyxa tunisiensis* are basal Ceratomyxidae for the phylogenetic arrangement of this family, and as demonstrated in the phylogenetic tree (Figure 3), these *Ceratomyxa* species mentioned above were found in the same clade (A) as *Ceratomyxa affinis* n. sp., corroborating the authors. These authors state that the long arms of the phylogenetic trees of Ceratomyxidae indicate rapid evolution of this group in freshwater environments.

*Ceratomyxa affinis* n. sp. behaved as an “outlier” species, due to its isolation in clade A1, that is, not grouping with any other *Ceratomyxa* species. Baum (2008) suggest that “outlier” species are closer to the root of the tree, indicating that they are considered to be the oldest or ancestral branches within the phylogenetic tree, or that they diverged earlier from the common ancestor of the group.

## Conclusions

Based on morpho-molecular analyses, it was possible to prove that *Ceratomyxa affinis* n. sp. is a new species. This contributes to knowledge of the parasitic fauna of fish in the Amazon region, in which this was the seventh species of this genus described in the state of Amapá, and the third described in fish from the municipality of Tartarugalzinho. That may indicate a rapid evolution of the Ceratomyxidae family in the Brazilian Amazon region.

## Data Availability

The DNA sequences are deposited in Genbank (PV765332); a glass slide with spores stained with hematoxylin and eosin (H&E) is deposited in the collection of the National Amazonas Research Institute (INPA), Manaus, Amazonas, Brazil (accession number: CND 000111). The data generated during the study are included in this article.
